# Allatostatin A Signalling: Progress and New Challenges From a Paradigmatic Pleiotropic Invertebrate Neuropeptide Family

**DOI:** 10.3389/fphys.2022.920529

**Published:** 2022-06-24

**Authors:** Christian Wegener, Jiangtian Chen

**Affiliations:** ^1^ Neurobiology and Genetics, Würzburg Insect Research, Theodor-Boveri-Institute, Biocenter, University of Würzburg, Würzburg, Germany; ^2^ Department of Ecology, Evolution and Organismal Biology, Brown University, Providence, RI, United States

**Keywords:** neuropeptide signalling, feeding, intestinal control, sleep/activity, kisspeptin/galanin/spexin signalling, metabolism and growth, learning, cardioactive factor

## Abstract

Neuropeptides have gained broad attraction in insect neuroscience and physiology, as new genetic tools are increasingly uncovering their wide-ranging pleiotropic functions with high cellular resolution. Allatostatin A (AstA) peptides constitute one of the best studied insect neuropeptide families. In insects and other panarthropods, AstA peptides qualify as brain-gut peptides and have regained attention with the discovery of their role in regulating feeding, growth, activity/sleep and learning. AstA receptor homologs are found throughout the protostomia and group with vertebrate somatostatin/galanin/kisspeptin receptors. In this review, we summarise the current knowledge on the evolution and the pleiotropic and cell-specific non-allatostatic functions of AstA. We speculate about the core functions of AstA signalling, and derive open questions and challengesfor future research on AstA and invertebrate neuropeptides in general.

## 1 Introduction

“What is Allatostatin (A) actually good for?” asked Leah.

“With regard to the cockroach or us?” asked Jean Ardley.

Carl Djerassi “Cantor’s dilemma,” 1989.

Four decades of research have revealed the outstanding importance of neuropeptides as context- and state-dependent intercellular messengers that help coordinate body functions at many levels: from modulation of small neuronal circuits to orchestration of different organs in the whole body and behaviour. Neuropeptide signalling is evolutionarily ancient, and neuropeptides represent the most diverse class of neuronal messengers.

The first insect and arthropod neuropeptides were chemically identified in the 1980ies, followed by an “explosion of structural information” in the 1990ies with the availability of sophisticated HPLC- and sequencing methods (Gäde, 1997). During that time, bioassays played an important role in identifying and purifying bioactive peptides, and the observed effects often led to the naming of the identified compounds. For instance, an *in vitro* bioassay for the production of juvenile hormone (JH) from the endocrine corpora allata of cockroaches led to the identification of peptides that were named allatostatins A (AstA) as they were the first peptides found to inhibit JH production and release (Pratt et al., 1989, 1991; Woodhead et al., 1989, 1994). In the new millennium, the availability of genomic/transcriptomic information and advanced mass-spectrometric techniques allowed to predict and biochemically confirm the whole peptidome of insect species. Combined with the development of increasingly sophisticated genetic tools, particularly for the fruit fly *Drosophila*, the field of insect neuropeptides is currently in an era facing an “explosion of functional information.”

Today, it is clear that AstA and many other insect neuropeptides have not just one but multiple pleiotropic functions that are dependent on the peptidergic cell type and internal and environmental context. Unfortunately, for AstA this is not reflected by its name based on the effect in the bioassay used during the initial identification. In fact, the allatostatic function of AstA appears to be restricted to hemimetabolous insects such as Orthoptera and Dictyoptera [see (Stay and Tobe, 2007)].

This review first takes a look at the sequence evolution of AstA peptides and their receptors, then continues with an overview of the pleiotropic functions of AstA across insects. We then propose ideas on how the diverse functions of AstA signalling can be functionally integrated and discuss possible ancient core functions. We conclude with a discussion on the questions that come along with the pleiotropic and cell- and context-specific functions of AstA and other neuropeptides, and how they could be experimentally addressed.

## 2 Evolution of AstA Peptides and Their G-Protein Coupled Receptors

**FIGURE 1:**
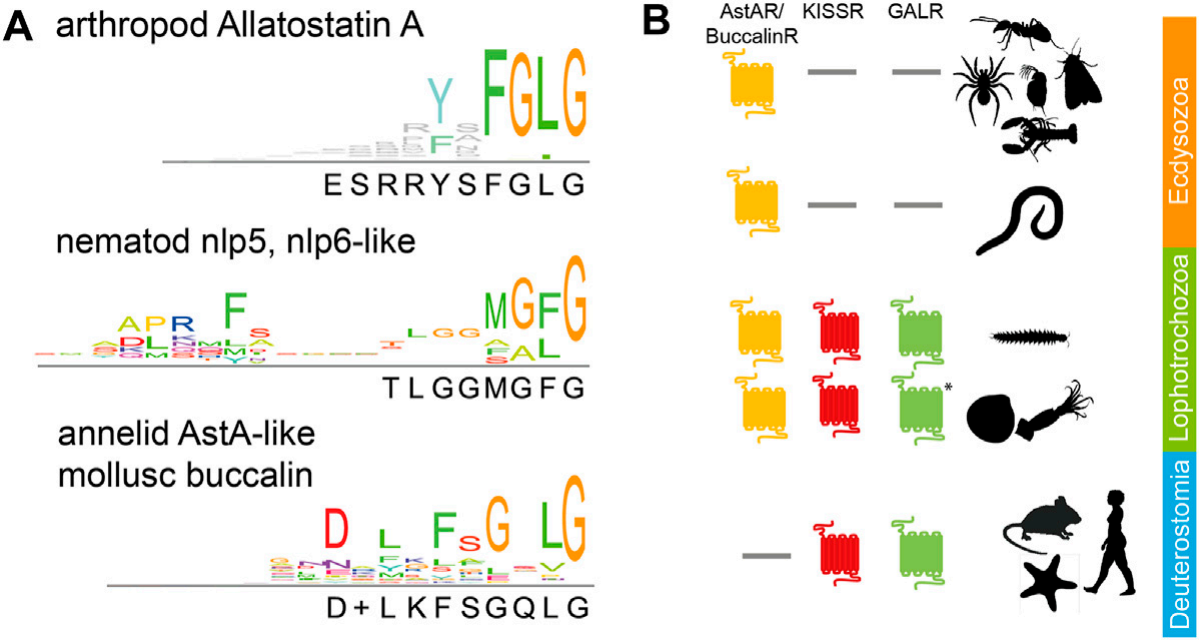
Phylogeny of AstA signalling: **(A)** Consensus sequences for AstA and similar peptides in arthropods (top), nematodes (middle), and lophotrochozoans (annelids, molluscs, bottom). The C-terminal glycine indicates the amidation site. Sequences are given in Supplementary Material 1; **(B)** While AstA-Rs are found throughout the protostomes, KISSRs, and GALRs have been lost in the Ecdysozoa [incl. Nematoda and Panarthropoda (Felix et al., 2015; Li et al., 2021)]. * = GALR-like receptors have only been found in cephalopods. Pictogrammes from www.phylopic.org.

AstA peptides are characterised by the C-terminal sequence Y/FXFGLamide, which is highly conserved throughout the arthropods, including Chelicerata and Mandibulata (Figure 1; Supplementary Material S1). AstA peptides should not be confused with Allatostatins B, C, CC, or CCC [see (Bendena and Tobe, 2012; Veenstra, 2016; Nässel and Zandawala, 2019)]. These distinct Ast peptide families neither share functional nor sequence homology with AstA.

There is one AstA prepropeptide-encoding gene in the analysed arthropod genomes, except for beetles that despite some evidence (Elliott et al., 2010) seem to entirely lack *AstA* based on genomic (Li et al., 2008; Felix et al., 2015) and peptidomic data (Li et al., 2008; Weaver and Audsley, 2008; Ragionieri and Predel, 2020). AstA prepropeptides contain from four [e.g., *Drosophila* (Lenz et al., 2000b)] to over 20 [e.g., lobster (Christie et al., 2015)] AstA paracopies. Available EST data reveal canonical Y/FXFGLamides also for tardigrades (Christie et al., 2011; Koziol, 2018) but not for onychophorans [(Christie et al., 2011) and own BLAST searches]. Yet, genomic information is not complete for onychophorans (G. Mayer, pers. commun.), and the expression of distinct AstA-immunoreactivity in their ventral nerve cords (Martin et al., 2017) suggests that also velvet worms possess AstA peptides. Outside the panarthropods, canonical Y/FXFGLamides have not been found. Within the Ecdysozoa, the nlp5 and nlp6-like peptides (MG/AF/Lamide, Figure 1) of nematodes show structural similarity (Nathoo et al., 2001; Husson et al., 2005). They may indeed represent AstA orthologs as there is an astonishingly conserved gene synteny in the genomic region surrounding the nlp5/6 genes of *C. elegans* and *AstA* in insects (Felix et al., 2015). In lophotrochozoans, the mollusc buccalins (Veenstra, 2010; Cardoso et al., 2016) and AstA-like peptides ending with Lamide in Platyhelminthes (McVeigh et al., 2009) and annelids (Conzelmann et al., 2013) (Figure 1) are considered to be AstA orthologs (Li et al., 2021). Yet, clear molecular and functional evidence is lacking. A C-terminal sequence similarity exists between AstA and vertebrate kisspeptins (consensus sequence NXFGLRYamide). A common phylogenetic origin of AstA and vertebrate kisspeptins is further suggested by gene synteny in the genomic neighbourhood of AstA and kisspeptin genes (Felix et al., 2015). However, direct homologisation is difficult since the genomic region around vertebrate kisspeptin contains genes for two closely related peptides [galanin and spexin (Kim et al., 2014)].

The first AstA receptors, AstA-R1 and AstA-R2, were characterised and deorphanised in the fruit fly *Drosophila melanogaster* (Birgül et al., 1999; Lenz et al., 2000a; Lenz et al., 2000b; Lenz et al., 2001; Larsen et al., 2001). The initial identification of AstA-R1 by RT-PCR used degenerate primers based on mammalian somatostatin receptors (SSTRs) (Birgül et al., 1999). However, the highest sequence similarity was found to mammalian galanin receptors [GALR, (Lenz et al., 2000a; Lenz et al., 2000b)], further members of the vertebrate SST/galanin/opioid family of GPCRs. The close relationship of AstA-Rs with GAL-Rs is confirmed by more comprehensive sequence and structural analyses (Jékely, 2013; Mirabeau and Joly, 2013; Urlacher et al., 2016; Li et al., 2021). Collectively, these data suggest that an ancestral receptor gene duplicated twice to give rise to the ancestral AstA-R/Buccalin-R, KISS-R, and GAL-R families prior to the divergence of proto- and deuterostomia (Felix et al., 2015; Li et al., 2021). This relationship of the receptors is matched by their related ligands (AstA, galanin, kisspeptin, and spexin) as outlined above. Both KISSR and GALR got lost in the Ecdysozoa, including Panarthropoda, while AstA-R got lost in Deuterostomia before the split of echinoderms (Figure 1B).

Two AstA-R genes is a typical feature of Diptera that was also found in the kissing bug *R. prolixus* (Zandawala and Orchard, 2015; Ons et al., 2016). Other insects appear to have only one AstA-R (Felix et al., 2015), including other Heteroptera (Benoit et al., 2016). Only in beetles, AstA-Rs are missing (Felix et al., 2015), matching the lack of an *AstA* gene in this taxon. The number of predicted AstA-R in crustacean genomes typically is one [e.g., in decapods (Christie et al., 2015; Dickinson et al., 2019; Tran et al., 2019; Christie, 2020) and copepods (Christie et al., 2013)]. Three AstA-R are predicted for the ctenopod water flea *Daphnia pulex* (Felix et al., 2015), and three to six AstA-R are predicted for tardigrades (Koziol, 2018). One [spider mite *Tetranychus urticae* (Veenstra et al., 2012)] and four [tick *Ixodes scapularis* (Felix et al., 2015)] AstA-Rs are predicted from available chelicerate genomes.

Collectively, the available sequence information indicates that AstA signalling is a common feature of Panarthropoda, with the notable exception of the beetles. While canonical AstA peptides are restricted to Panarthropoda, AstA-Rs occur throughout the protostomes to hemichordates, with AstA signalling phylogenetically closely related to kisspeptin and galanin signalling.

## 3 Non-Allatostatic Functions of AstA Signalling in Insects

### 3.1 AstA Functions in the Periphery

#### 3.1.1 Regulation of Gut Physiology and Senescence

Early research in cockroaches showed that the rectal dilator muscle and the muscles of the rectum and anterior hindgut are innervated *via* AstA-immunopositive (AstA^+^) fibres of the proctodeal nerve (Lange et al., 1993; Yu et al., 1995). AstA indirectly modulates foregut movements *via* AstA^+^ neurons in the tritocerebrum, which project into the stomatogastric nervous system (Maestro et al., 1998). Moreover, synthetic AstA inhibited spontaneous hindgut (Lange et al., 1993; Lange, 1995) and proctolin-induced midgut contractions (Fusé et al., 1999). AstA was further found to be produced by enteroendocrine cells and classified as brain-gut peptides in cockroaches (Reichwald et al., 1994; Yu et al., 1995). To date, results from various groups suggest three shared features across insects, except beetles: AstA^+^ innervation of the hindgut, expression of AstA in enteroendocrine cells of the midgut (Wegener and Veenstra, 2015) and an inhibitory effect of AstA on gut peristalsis (Supplementary Material S2). In the midgut, AstA regulates the release of digestive enzymes (amylase, invertase, and proteases) in various insects (Supplementary Material S3). In locusts and moths, AstA^+^ neurons are also found in the frontal ganglion (Duve et al., 2000; Robertson and Lange, 2010; Roller et al., 2016), a part of the stomatogastric nervous system and central pattern generator of foregut peristalsis in many insects. In locusts, AstA inhibits foregut contractions, decreases the bursting cycle of the central pattern generator output of the frontal and ventricular ganglion, and disrupts rhythmicity at higher concentrations (Zilberstein et al., 2004; Robertson et al., 2012).

A direct role of AstA in processing ingested food is further substantiated by a significant reduction of AstA^+^ EECs in the midgut of *S. littoralis* upon starvation (Nakhaie Bahrami et al., 2018). While AstA immunoreactivity in the EECs was unchanged between starved and blood-fed kissing bugs *R. prolixus*, AstA is released from neuronal sources in this species (Zandawala and Orchard, 2013).

AstA further controls K^+^ ion transport across the mid- and hindgut. In the anterior mosquito midgut, AstA induced decreases in the transepithelial voltage and subsequently diminished transepithelial ion transport (Onken et al., 2004). In *Drosophila* larvae, AstA slightly increased K^+^ absorption in the anterior midgut and significantly decreased K^+^ absorption in the region of the middle midgut copper and large flat cells and the neutral zone of the posterior midgut. In the ileum, AstA induced K^+^ secretion into the lumen, while the addition of saline and other peptides reversed the direction and led to K^+^ absorption (Vanderveken and O’Donnell, 2014). In locusts and larvae of the midge *Chironomus riparius*, AstA decreased K^+^ absorption in the anterior ileum (Robertson et al., 2014b) or rectum (Robertson et al., 2014a), respectively.

Gut-derived AstA may also be involved in pathogen defence. The number of AstA^+^ EECs in *Drosophila* is upregulated upon infection with the pathogenic Gram-negative bacterium *Pseudomonas entomophila* (Beebe et al., 2015). AstA also appears to delay midgut senescence by inhibiting the proliferation of intestinal stem cells (ISCs) (Takeda et al., 2018). Preferential downregulation of AstA in EEs resulted in earlier onset of ISC-like tumours, a marker of midgut senescence. This effect is likely due to an AstA-R1-mediated direct action of AstA on ISCs or enteroblasts (Takeda et al., 2018). How AstA signalling *via* AstA-R1 delays molecular senescence signalling pathways remains to be clarified.

**FIGURE 2:**
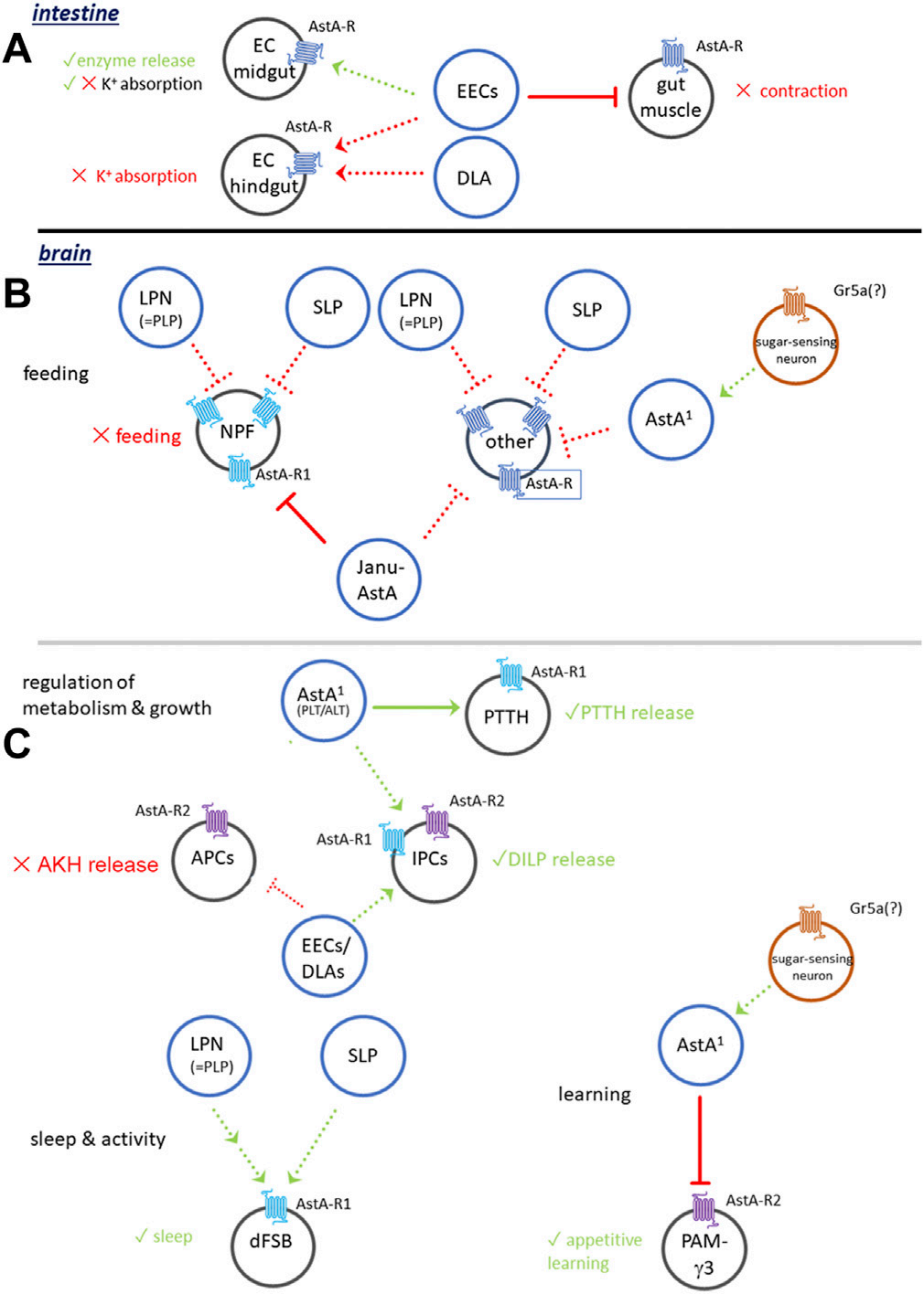
Generalised model of AstA signalling in *Drosophila*. **(A)** Functions in the intestinal tract. **(B)** AstA signalling in the regulation of feeding. **(C)** AstA signalling in the regulation of metabolism and growth, sleep and activity and learning. For details see Section 3.1.1, Section 3.2. Dashed connections are hypothetical.

Taken together, these data imply a variety of gut-related functions of AstA locally released by midgut EEs or neuronal endings at the posterior midgut and hindgut. Once released, AstA inhibits gut motility and K^+^ absorption in the posterior midgut and hindgut and activates the release of digestive enzymes across the investigated insect species (Figure 2A). This suggests a post-prandial activity of AstA signalling in the gut and a core function of AstA in facilitating increased and prolonged food digestion. The reported massive release of AstA from abdominal neurohaemal release sites 1–5 h following a blood meal in the kissing bug *R. prolixus* (Zandawala and Orchard, 2013) supports this idea.

In line with a post-prandial release, the effect on transepithelial K^+^ transport likely serves to deal with an excess of K^+^ ions and fluid gained from a recent meal (Robertson et al., 2014b), though AstA appears neither to have diuretic/antidiuretic functions nor to act at the Malpighian tubules. The physiological significance of this regulation on the level of the intestinal tract is indicated by a senescence-independent decrease in life span upon a midgut-preferential down-regulation of AstA (Takeda et al., 2018), though other effects (see below) may also be involved.

#### 3.1.2 Regulation of Cardioactivity

A recent screen for cardioactive peptides in the *Drosophila* larva revealed a role of AstA in modulating heart rate and rhythmicity (Schiemann et al., 2019). When applied to a semi-intact larval preparation, AstA had a mild to strong chronotropic effect and at 10^−7^ M led to heartbeat arrest. In contrast, *in vivo* RNAi-mediated systemic AstA knock-down resulted in a significantly increased heart rate and decreased rhythmicity (Schiemann et al., 2019). In insects, the heartbeat is myogenic, and the heart does not receive innervation. It seems possible that the opposed effects of AstA between semi-intact preparations and intact larvae are due to an inhibitory effect of AstA on the release or the muscular action of chronotropic cardioactive peptides. This is suggested by findings from the antennal heart of the cockroach *Periplaneta americana*, in which AstA has no direct cardioactive effect but inhibits the cardioacceleratory effects of the peptide proctolin (Hertel and Penzlin, 1992). In the German cockroach (*Blattella germanica*), however, AstA strongly reduces the cardiac rhythm in a semi-intact heart preparation, suggesting a direct cardioinhibitory function (Vilaplana et al., 1999). In tenebrionid beetles, AstA lacks cardioactivity (Lubawy et al., 2018) in line with the general lack of AstA signalling in this order.

### 3.2 Central Actions of AstA

**FIGURE 3:**
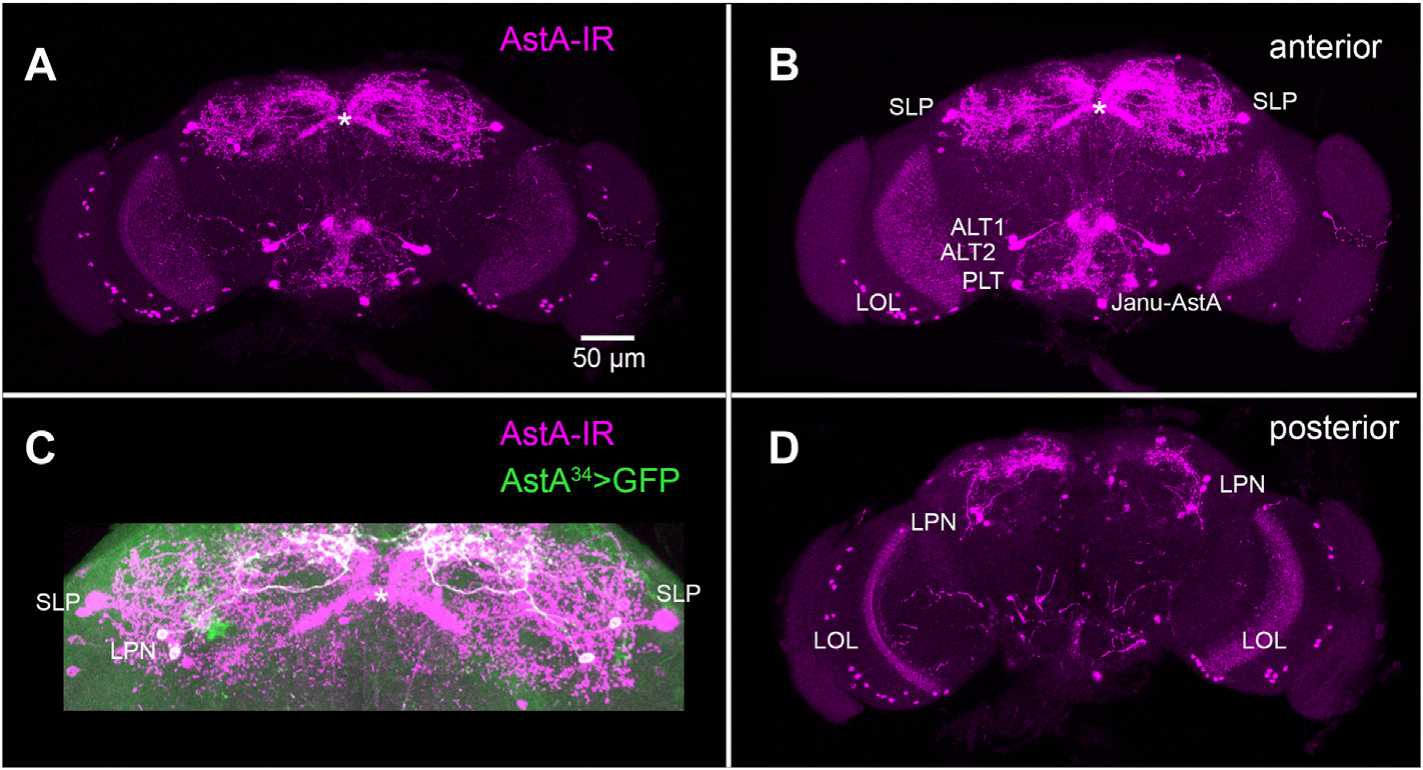
AstA neurons in the adult fruit fly brain. **(A)** Maximum projection of a whole mount adult brain stained with an antiserum against Dippu-AstA-7 [(Vitzthum et al., 1996), magenta]. **(B)** Stack of the anterior optical sections of the brain in **(A)**. **(C)** Superior region of the protocerebrum from **(A)**, showing additional labelling of AstA^34^ > GFP expression (green) which marks the LPNs. It is visible that the LPNs do not innervate the fan-shaped body of the central complex [asterisk, (Reinhard et al., 2022)]. **(D)** Stack of the posterior optical sections of the brain in **(A)**. Original preparations. Scale bar = 50 µm is identical for **(A–D)**.

As a typical brain-gut peptide, AstA is not only expressed in the gut but also in a considerable number of neurons in the central nervous system (CNS) of insects. Excitingly, the recent availability of restricted Gal4-driver lines in *Drosophila* has opened a cell type-specific perspective on AstA signalling. This will be discussed below and requires a brief description of the cellular expression pattern of AstA in *Drosophila*. The fly CNS contains around 102 AstA-immunoreactive neurons (Yoon and Stay, 1995) (Figure 3A). These include per hemisphere: around 30 lateral optic lobe cells (LOL, Figures 3B,D), two superior-lateral protocerebral neurons (SLPs) (Figures 3B,C), three lateroposterior protocerebral neurons (LPNs, Figures 3C,D), two anterior-lateral tritocerebral neurons (ALT1 and ALT2, Figure 3B), one posterio-lateral tritocerebral neuron (PLT, Figure 3B), one ventro-medial neuron with soma in the gnathal ganglion (VMG, or Janu-AstA, Figure 3B), and three small dorsolateral abdominal (DLAa) neurons which innervate the hindgut (Yoon and Stay, 1995; Hergarden et al., 2012; Chen et al., 2016; Landayan et al., 2021). Outside of the CNS, AstA is expressed in two pairs of peripheral AstA-immunoreactive neurons with somata on the segmental nerves and projections to the wings and halteres (Yoon and Stay, 1995).

#### 3.2.1 Regulation of Food and Water Intake

Inhibition of feeding seems to be a common function of AstA. For instance, injection of synthetic AstAs into the cockroach *Blattella germanica* reduced food uptake by 50%–60% (Aguilar et al., 2003). Detailed knowledge of the relation between AstA signalling and feeding comes from recent work in genetically amenable *Drosophila*.

Genetic activation of AstA^+^ LPNs and the EECs leads to a robust and significant reduction of feeding (Hergarden et al., 2012; Chen et al., 2016). This effect disappeared in an AstA mutant background (Chen et al., 2016), demonstrating that it depends on AstA signalling.

The ventromedian pair of AstA^+^ neurons in the GNG [VMG (Yoon and Stay, 1995) or Janu-AstA (Landayan et al., 2021) neurons] inhibits feeding on dry yeast in a binary choice between dry yeast and water (Landayan et al., 2021). A major function of the Janu-AstA cells is to promote water seeking in water-repleted flies (Landayan et al., 2021), and more research is required to find out whether the altered binary choice represents genuine modulation of feeding. Importantly, Janu-AstA neurons do not seem to regulate water intake per se (Landayan et al., 2021). Similar to the AstA^+^ LPN and SLP neurons (Yoon and Stay, 1995; Chen et al., 2016; Ni et al., 2019), also the Janu-AstA neurons send projections to the superior median protocerebrum [SMP, (Landayan et al., 2021)] which appears as a key area that integrates AstA signalling in the regulation of feeding and water seeking. In the SMP, Janu-AstA projections seem to be presynaptic to NPF neurons (Landayan et al., 2021). RNAi-mediated knock-down of AstA-R2 (but not AstA-R1) in NPF neurons decreased water-seeking and increased feeding behaviour in thirsty flies. The AstA^1^-Gal4 line (Hergarden et al., 2012) expresses in many LOL, the LPN, VMG/Janu-AstA and DLAa neurons, the two pairs of AstA-immunoreactive neurons on the segmental nerves, as well as many AstA+ enteroendocrine cells (Hergarden et al., 2012; Chen et al., 2016). Interestingly, NaChBac-mediated co-activation of AstA^1^ (including the Janu-AstA neurons) and NPF neurons leads to a feeding rate roughly intermediate to activation of either AstA^1^ (decreased feeding) or NPF (increased feeding) (Hergarden et al., 2012). Figure 2B provides a generalised summary of AstA signalling in the regulation of feeding and water seeking.

Activation of AstA^1^ neurons by NaChBac strongly suppressed the starvation-induced enhancement of the proboscis extension reflex (PER) and intake of a concentrated sucrose food solution in *Drosophila* (Hergarden et al., 2012). In contrast, PER was unaltered in fed flies (Hergarden et al., 2012). Hence, the inhibitory effects of AstA^1^ cell activation on feeding and PER are not due to impaired gustatory sensing or reduced capability to feed. In line, RNAi-mediated downregulation of AstA in AstA^1^ cells did not alter the preference to 2M sucrose (Yamagata et al., 2016). AstA^1^>NaChBac flies showed a significantly reduced sugar-over-yeast preference (males) or a clear yeast-over-sugar preference (females) compared to controls of both sexes which equally and strongly preferred sucrose over yeast (Hentze et al., 2015). Concomitantly, AstA mutant flies consumed significantly more sucrose than controls in a capillary feeder assay (Hentze et al., 2015). These findings suggest that activation of AstA^1^ cells signals carbohydrates or carbohydrate satiety and shifts the food preference away from sugar towards protein. Interestingly, AstA^+^ arborisations in the GNG are in close contact with Gr5a-expressing sucrose-sensing gustatory neurons (Hergarden et al., 2012). Yet, a functional connection between the Gr5a and AstA+ neurons still needs to be demonstrated.

In honey bees starved for a short period (1 h), synthetic AstA injected into the brain did not alter the PER response to increasing sucrose concentration (Urlacher et al., 2016). Also, olfactory discrimination in a generalisation learning paradigm was unaltered in honey bees upon AstA injection into the brain (Urlacher et al., 2016).

#### 3.2.2 Regulation of Metabolism

Several findings argue against a direct metabolic action of AstA. Despite the significant feeding suppressant effect of AstA in *Drosophila* discussed above, constitutive activation of AstA cells did not affect whole-body triglyceride and glucose levels when flies were fed *ad libitum* (Hergarden et al., 2012). Also, upon starvation, AstA^1^>NaChBac flies depleted triglyceride depots similar to controls and showed comparable starvation resistance (Hergarden et al., 2012). Triglyceride stores were also unaffected in *Drosophila* larvae with a global RNAi-mediated knock-down of AstA-R1 or AstA transcripts (Wang et al., 2012).

Nevertheless, AstA is one out of several peptides that modulate the endocrine centres controlling carbohydrate and lipid metabolism: the *Drosophila* insulin-like peptides (DILP)-producing cells (IPCs) in the pars intercerebralis and the adipokinetic hormone (AKH)-producing cells (APCs) in the glandular part of the *corpora cardiaca* (Hentze et al., 2015). Both APCs and IPCs express the AstA-R2 receptor, as indicated by immunostainings and AstA-R2-specific Gal4-driven GFP expression. AstA-R2 downregulation in the APCs and IPCs increased starvation resistance, which is compatible with the feeding-inhibitory role of AstA signalling (Hentze et al., 2015). Downregulation of AstA-R2 in the IPCs also induced a strong reduction in *dilp2* expression in females but not males, while *dilp3* expression was unaffected. Functional silencing of AstA^1^ cells led to an upregulation of AKH receptors (AKHR), no change in *Akh* expression, a moderate upregulation of *dilp2* and a strong reduction in *dilp3* transcription. AstA^1^>NaChBac activation did not affect *Akh* expression, but promoted *dilp2* and especially *dilp3* expression (Hentze et al., 2015). In *Drosophila* larvae, the IPCs express AstA-R1, and RNAi-mediated downregulation results in increased immunostaining intensity and decreased transcription of *dilp2* and *dilp5* in the IPCs (Deveci et al., 2019). This points towards reduced DILP release, supported by significantly reduced haemolymph titres of DILP2 and a reduced growth rate when AstA signalling was downregulated in *Drosophila* larvae (Deveci et al., 2019).

Collectively, the available evidence suggests that AstA is able to promote DILP release from the IPCs and to inhibit AKH release from the APCs. This is compatible with the suggested role of AstA in feeding-inhibition and carbohydrate signalling. In fact, the system seems to be activated by carbohydrates, as a restricted diet of 1% sucrose leads to a significant global downregulation of AstA and AstA-R2 transcripts (Hentze et al., 2015), while refeeding with a carbohydrate-rich, but not protein-rich diet strongly increased the expression (Hentze et al., 2015). Feeding casein peptidone and amino acids largely failed to activate AstA-expressing EECs in the posterior midgut of *Drosophila* (Park et al., 2016).

Constitutive activation of AstA^1^-cells resulted in transcriptional upregulation of *tobi* [*target of grain insulin* (Buch et al., 2008; Hentze et al., 2015)], coding for an α-glucosidase downstream of AKH and insulin signalling (Buch et al., 2008). This effect may well be due to increased insulin secretion or decreased feeding upon increased AstA signalling. In line, RNAi-mediated knock-down of AstA-R2 in IPCs induced a downregulation of *tobi* expression particularly in males. Furthermore, AstA mutant flies showed a drastic reduction in *tobi* expression, and increased size of lipid droplets and starvation resistance (Hentze et al., 2015) similar to flies lacking APCs or IPCs (Lee and Park, 2004; Broughton et al., 2005; Bharucha et al., 2008). This likely is a consequence of reduced insulin signalling and increased feeding upon reduced AstA signalling.

Taken together, AstA may be part of a carbohydrate sensing mechanism that helps keep the balance of IPC-APC signalling (Hentze et al., 2015) and shifts food intake from carbohydrates to proteins (see above). In *Drosophila*, the corpora cardiaca containing the APCs are not innervated by AstA neurons (Yoon and Stay, 1995). Therefore, AstA acting on APCs is most likely hormonally released from AstA-expressing EECs (Hentze et al., 2015), the peripheral neurons, or the abdominal AstA neurons innervating the hindgut.

In *Locusta migratoria*, AstA appears to positively modulate AKH signalling, pointing towards a conserved modulatory function across the insects. In this locust, the corpora cardiaca are innervated by AstA^+^ neurosecretory cells with somata in the lateral protocerebrum (Clark et al., 2008). Synthetic AstA significantly increased the release of AKH I and elevated the cAMP content from isolated glandular corpora cardiaca lobes (Clark et al., 2008).

#### 3.2.3 Regulation of Growth

The prothoracicotropic hormone (PTTH) neurons are a key target of AstA signalling related to growth. In *Drosophila*, the PTTH neurons time development and influence body size by the PTTH-dependent regulation of steroid hormone (ecdysone) production in the prothoracic gland (McBrayer et al., 2007). In the larva, the PTTH neurons express AstA-R1 (Deveci et al., 2019). Downregulation of AstA-R1 or electrical silencing of the PTTH neurons resulted in more and enlarged PTTH-containing varicosities at the release sites of the prothoracic gland. It also lowered the PTTH titre in the haemolymph and significantly extended the larval stage by around 20 h, resulting in delayed pupariation (Deveci et al., 2019). The lengthening of the larval stage allows for a longer time of larval growth, resulting in around 10% larger pupae (Deveci et al., 2019). AstA null mutants are approximately 10% smaller, while RNAi-mediated downregulation in all AstA cells delayed the time to pupariate but did not affect growth.

These results suggest a modulatory role of AstA on growth and development. The likely source of AstA that modulates larval PTTH neurons are cell groups in the lateral tritocerebrum/GNG [named ALT and PLT (Yoon and Stay, 1995), or N1 and N2 (Deveci et al., 2019)]. Electrical silencing of these neurons delayed pupariation while the pupal size remained normal. The same was also observed when electrical silencing or AstA-RNAi was restricted to the ALT/N1 neurons. Thermogenetic activation of PLT/N2 neurons led to a slightly earlier pupariation without affecting growth (Deveci et al., 2019). Collectively, this indicates a functional connection of the larval N1 (and perhaps also N2) to PTTH neurons and the IPCs. Both targets seem to be activated by AstA-AstA-R1 signalling: blocking AstA signalling to the PTTH neurons delayed development [similar to negatively manipulating PTTH signalling (McBrayer et al., 2007)], and blocking AstA signalling to the IPCs slowed down growth rate (IPCs are main regulators of growth [see (Koyama et al., 2020)]. AstA therefore appears as an important modulatory factor in balancing growth and developmental timing, again with a positive modulation of regulator release (PTTH, DILPs, Figure 2C).

#### 3.2.4 Modulation of Foraging, Locomotor Activity, and Sleep

The first evidence of an effect of AstA on locomotor activity was obtained in *Drosophila* larvae. Global downregulation of either AstA or AstA-R1 caused a significantly reduced path length on a small layer of yeast (Wang et al., 2012). This difference disappeared when larvae were tested off food, suggesting that AstA influences foraging (food search) behaviour (Wang et al., 2012). This effect is reminiscent of the sitter/rover phenotype, which differs in the activity of Foraging (FOR), a protein kinase G (Osborne et al., 1997). Interestingly, global downregulation of AstA or AstA-R1 reduced the transcript levels of FOR in *Drosophila* larvae (Wang et al., 2012). This suggests a functional connection between AstA signalling, FOR and the sitter/rover phenotype, which yet is to be demonstrated. Injection of synthetic AstA into the mosquito *Aedes aegypti* significantly reduced host-seeking behaviour (i.e., foraging), an effect that was additive to the host-seeking inhibition by short neuropeptide F (sNPF) (Christ et al., 2017). Part of this effect might be mediated by altered AstA modulation of olfactory information processing in the mosquito antennal lobe, in which blood only or blood and sugar-feeding led to significantly lower levels of AstA compared to sugar only feeding (Christ et al., 2017).

The general locomotor activity in starved or fed adult *Drosophila* was unaffected by constitutive UAS-NaChBac-mediated activation of AstA^1^ cells (Hergarden et al., 2012). However, in both sexes, conditional thermogenetic activated LPNs and EECs *via* a more restricted AstA^34^-Gal4 driver line significantly reduced locomotor activity without affecting circadian rhythmicity and promoted sleep, especially during the flies’ morning and evening activity peaks (Chen et al., 2016). Conditional but not constitutive silencing of LPNs and EECs reduced sleep, especially during the midday siesta and significantly increased total locomotor activity (Chen et al., 2016). In line, conditional activation of AstA cells significantly increased the arousal threshold, especially during evening peak activity (Chen et al., 2016). AstA signalling seems essential for these sleep-promoting effects since thermogenetic activation of LPNs and EECs in an AstA null mutant background failed to alter sleep and locomotor activity (Chen et al., 2016).

Interestingly, the AstA^+^ LPN neurons are part of the central circadian clock network in *Drosophila* (Guo et al., 2018; Ni et al., 2019; Reinhard et al., 2022). They are in close contact with the dorsal terminals of the small ventral lateral neurons (sLNvs), another group of circadian clock neurons expressing the peptide pigment-dispersing factor (PDF) (Chen et al., 2016). Expression of a genetic marker for the PDF receptor (PDFR) and cAMP imaging indicate that the LPN neurons are targets of circadian PDF signalling from the sLNvs. These findings suggest that the circadian clock *via* PDF signalling from the sLNvs might modulate LPN neuron activity to promote sleep. In line with this idea, expression of tethered PDF in AstA^34^ cells as well as thermogenetic activation of sLNvs significantly promoted sleep (Chen et al., 2016). Also, genetic activation of a small subset of clock neurons including the LPNs or only the LPNs strongly promoted sleep (Guo et al., 2018; Ni et al., 2019), an effect that was reduced in an AstA mutant background (Ni et al., 2019).

Besides the LPNs, the AstA^+^ SLP neurons are also able to promote sleep (Ni et al., 2019). Co-activation of SLP and LPN neurons *via* NaChBac in an AstA mutant background still resulted in prolonged sleep and longer sleep bouts during the photoperiod compared to activation of the SLP neurons alone (Ni et al., 2019). The combined effect was especially prominent during the second half of the light phase when also thermogenetic activation of LPN neurons had the strongest effect (Chen et al., 2016). Taken together, these results indicate a sleep-promoting role of AstA and co-localised glutamate from the LPN and SLP neurons (Ni et al., 2019).

The central complex is a crucial structure controlling sleep-wake balance in the insect brain. The neuronal circuitry for sleep regulation in the central complex comprises an interconnected network of dorsal fan-shaped body (dFSB) neurons, so-called helicon cells and R5 neurons (Suárez-Grimalt and Raccuglia, 2021). The dFSB neurons seem to function as a master switch because their activation reduces arousal and induces sleep (Suárez-Grimalt and Raccuglia, 2021). Several lines of evidence suggest that these neurons are downstream targets of AstA signalling. First, the dFSB neurons are marked by an AstA-R1 reporter line [23E10-Gal4 (Donlea et al., 2018)]. Second, the LPNs make synaptic contacts to the dFSB and the mushroom body (Ni et al., 2019; Reinhard et al., 2022), another sleep regulatory region. GFP reconstitution across synaptic partners (GRASP) indicates close contact between the AstA^+^ LPN and SLP neurons in the superior median protocerebrum. Activation of the LPN and SLP neurons leads to an increased Ca^2+^ signal in the dFSB in line with the sleep-promoting effect of AstA and the dFSB neurons (Ni et al., 2019). Third, layer five of the fan-shaped body is strongly AstA-immunoreactive (Kahsai and Winther, 2010) and contains arborisations of the 23E10-Gal4 dFSB neurons (Donlea et al., 2018). RNAi-mediated AstA knock-down in the 23E10-Gal4 neurons reduced basal sleep and eliminated sleep-rebound after sleep deprivation. It was first suggested that dFSB neurons themselves might express AstA (Donlea et al., 2018). Yet, the 23E10-Gal4 labels several cells outside dFSB neurons, and these may include LPN or SLP neurons.

Taken together, these findings suggest that AstA signalling promotes sleep and does so most efficiently in the second half of the photoperiod. AstA is released by circadian clock neurons (LPNs) as well as SLP neurons, activating AstA-R1-expressing “sleep-inducing” dFSB neurons in the superior median protocerebrum. The origin of the AstA-immunoreactivity in layer five of the fan-shaped body has not been conclusively demonstrated. Yet, based on immunostainings, it is possible that this innervation is derived from SLP neurons (Figures 3A–C). AstA from the LPNs seems to provide circadian input to modulate activity during the morning and evening activity bouts (Chen et al., 2016; Ni et al., 2019).

However, the picture may be more complex than this and needs further research. First, a recent pilot screen for sleep-regulatory genes found that a knock-out of either AstA or one of the AstA receptors significantly increased both day- and night-time sleep (Deng et al., 2019). This may suggest overlapping and opposing cell-type specific AstA effects on sleep. Second, a recent study using an LPN-specific split-Gal4 line did only find weak (and in part opposing) effects on the activity and sleep pattern upon constitutive LPN ablation or conditional thermogenetic activation, though the reported sleep phenotype of the AstA^34^ neuron manipulations was reproduced (Reinhard et al., 2022). These results may point towards a role of hormonally released AstA from the EECs in the regulation of sleep and activity patterns.

#### 3.2.5 Modulation of Learning

Research in *Drosophila* and honey bees suggests a role for AstA in appetitive olfactory learning. AstA and the AstA-R are widely expressed in the brain in the honey bee, with a particular connection to the mushroom bodies (Kreissl et al., 2010; Urlacher et al., 2016), essential centres for olfactory learning. Injection of synthetic AstA into the head capsule of honey bees produced significantly reduced conditioned responses during appetitive olfactory learning (Urlacher et al., 2016). In *Drosophila*, a block of AstA^1^ neurons by UAS-shibire led to significantly decreased appetitive odour memory when sucrose or arabinose was used as the unconditioned stimulus (US) (Yamagata et al., 2016). In line, thermogenetic activation of AstA^1^ neurons as a substitute for the US (sugar) induced significant appetitive odour memory (Yamagata et al., 2016). These effects are mediated by AstA, as RNAi-mediated downregulation of AstA or CRISPR/Cas9-generated AstA null alleles resulted in significantly impaired appetitive learning (Yamagata et al., 2016). The fly AstA^1^ neurons exert their effect on appetitive learning by an AstA-R1-dependent inhibition of so-called protocerebral anterior medial (PAM)-γ3 neurons. These PAM-γ3 neurons signal *via* dopamine and mediate sugar reward when their activity is suppressed (Yamagata et al., 2016).

These results seem to suggest that the sign of the effect of AstA on appetitive olfactory learning is reversed between honey bees and fruit flies. Though purely speculative, the signalling pathway from AstA to PAM neurons may provide an explanation. Generally, dopamine is seen as a reward signal. Different sets of dopaminergic PAM cluster neurons signal reward when activated (Yamagata et al., 2015; Rohwedder et al., 2016) in contrast to the rather unusual PAM-γ3 neurons, which signal reward when inactive. Thus, in the honey bee, it might be possible that sugar activates AstA neurons that inhibit dopaminergic neurons that signal reward to the mushroom body when active. In contrast, the model derived from the results in *Drosophila* (Figure 2C) suggests that sugar (US) activates AstA^1^ neurons which via AstA-AstA-R1 inhibit the activity of PAM-γ3 which then signal reward.

## 4 Is There an Overarching Function of AstA Signalling in Insects?

A canonical function of neuropeptides is to coordinate and orchestrate different tissues across the body within a specific physiological context. If the core functions of AstA signalling are the regulation of digestion, sleep/activity, foraging, and appetitive learning, then it is tempting to speculate that the overarching systemic function of AstA signalling is to set the animal into a digestive/anabolic state balanced with circadian and developmental timing to optimise growth and reproduction. Also, the allatostatic effect of AstA (Stay and Tobe, 2007) would fit well with this idea. At least, AstA is unlikely to act as a simple “rest-and-digest” peptide as it has no effect on short-term post-prandial sleep [personal comm. William Ja, (Murphy et al., 2016)]. Furthermore, AstA signalling in the different signalling modules (see Figure 2) might be independent of each other. The widespread arborisations of AstA neurons (Figure 3) and broad expression of AstA receptors (Kondo et al., 2020) indicate that further new functions of AstA signalling are likely to be uncovered in the future (e.g., modulation of vision). Nevertheless, what seems clear is that AstA generally acts as an extrinsic modulator rather than as a direct signal required within, say, feeding, or sleep circuits.

## 5 Are Core Functions of Insect AstA Signalling a Derived or Basal Feature?

One core function of AstA in insects is the EEC-derived regulation of gut physiology. Outside the insects, AstA is expressed in EECs of crustaceans (Yin et al., 2006) and nematodes (Nathoo et al., 2001), suggesting that enteroendocrine AstA may be a common feature of Ecdysozoans. As the insect hindgut, the hindgut in ticks is innervated by AstA^+^ neurons situated in the posterior end of the synganglion (Šimo and Park, 2014). AstA nevertheless has no effect on hindgut motility in the tick (Šimo and Park, 2014). In the crab *Cancer borealis*, AstA-positive fibres from the commissural ganglia and the lateral ventricular and medial gastric nerve innervate the stomatogastric ganglion (STG) (Skiebe and Schneider, 1994), which regulates gut movement by generating various motor output rhythms (Marder and Bucher, 2007). Within the STG, AstA has a modulatory function and slows down the frequency of the pyloric rhythm (Skiebe and Schneider, 1994). AstA further decreases the gain of neuromuscular cholinergic and glutamatergic transmission directly at stomatogastric muscles (Jorge-Rivera and Marder, 1997). It also increases the spike-timing precision of the AstA-expressing gastropyloric receptor 2 in *C. borealis* by decreasing the spike rate upon stretch-stimulation (Billimoria et al., 2006). Thus, AstA in crabs modulates the control of gut movement both centrally and in the periphery.

As reviewed above, there is some evidence for a cardioregulatory role of AstA in insects. Unlike in insects, heartbeat in crustaceans is neuronally controlled by the cardiac ganglion. In the crab *Cancer borealis,* AstA inhibits both burst frequency and spike number of motoneurons in the cardiac ganglion (Cruz-Bermúdez and Marder, 2007), similar to its action in the stomatogastric ganglion.

Findings from *Caenorhabditis elegans* support a core function of AstA-R signalling in foraging and feeding (Bendena et al., 2008). A mutation in the *C. elegans* neuropeptide receptor NPR-9, a homolog of AstA-R (Felix et al., 2015), leads to strongly reduced roaming on but not off food. This effect was rescued by transgenic overexpression of *npr-9,* which led to a mild hyperroaming phenotype (Bendena et al., 2008). This situation is reminiscent of the phenotype of *Drosophila* larvae with downregulated AstA-R1 signalling (Wang et al., 2012). Moreover, *npr-9* mutant worms showed increased lipid storage in the form of lipid droplets (Bendena et al., 2008), which could be due to a feeding-inhibitory effect of NPR-9 signalling or the decreased roaming, which allows more time to dwell.

Collectively, these results suggest that modulation of gut movement, heartbeat and foraging/feeding are conserved functions of AstA signalling in Panarthropoda and Ecdysozoa. In Lophotrochozoa, the molluscan AstA equivalent buccalin is also closely associated with feeding (Di Cosmo and Polese, 2013). In the sea hare *Aplysia*, buccalins are expressed in various types of neurons which innervate peripheral tissues associated with feeding, digestion (incl. the gut), circulation and reproductive systems (Miller et al., 1992). These neurons include the motoneurons B15 and 16 in the buccal ganglia that innervate the accessory radula closer (ARC) muscle, which is required for food intake (biting). Buccalins decrease the size of contractions of the ARC muscle, thereby reducing food intake (Cropper et al., 1988). Buccalin-immunoreactive neurons were also found to innervate the unique feeding structures in another sea slug, Clione limacina (Norekian and Satterlie, 1997), and are expressed in neurons of the central feeding circuit in the snail *Lymnaea stagnalis* (Santama et al., 1994). These results suggest that regulation of feeding and digestion may be a primary basal function of AstA/buccalin signalling.

Outside the insects, direct experimental evidence for a regulatory function of AstA in learning and memory, locomotor activity and sleep is missing. Yet, intense and layered AstA immunoreactivity is found in the central body of decapods [a part of the crustacean central complex (Dircksen et al., 1999; Utting et al., 2000; Krieger et al., 2012)]. In the spider Cupiennius salei, the arcuate body [corresponding to parts of the Pancrustacean central complex (Lehmann et al., 2016)] shows strong and layered AstA immunoreactivity (Loesel et al., 2011). Therefore, AstA may potentially modulate activity and sleep in these taxa similar to *Drosophila* (Suárez-Grimalt and Raccuglia, 2021).

## 6 Future Challenges

We reviewed the current knowledge on the pleiotropic and cell-specific functions of AstA signalling. Further functions are likely to be found for AstA peptides in the future. For instance, the abundance of AstA^+^ neurons or projections in the optic lobes of insects and crustaceans (Yoon and Stay, 1995; Vitzthum et al., 1996; Dircksen et al., 1999; Panchan et al., 2003; Sarkar et al., 2003; Kreissl et al., 2010; Krieger et al., 2012; Kress et al., 2016) suggests a function of AstA in modulating the processing of visual information which awaits demonstration. Further, the functions of peripheral AstA neurons remain to be characterised.

Derived from the knowledge reviewed above, we would like to point out a few generic challenges for research on AstA, that largely apply for other invertebrate peptides as well (Nässel et al., 2019):

1) AstA and many other neuropeptides have pleiotropic and cell-specific functions. This requires appropriate cell or subset-specific manipulations to avoid interference from overlapping functions that may mask individual functions in a compound phenotype. In *Drosophila*, this is possible by intersectional approaches (Luan et al., 2006). In larger insects, at least prominent neurosecretory cells may be ablated by microsurgery or laser ablation.
2) The widespread co-localisation of AstA with other neuropeptides or classic transmitters requires cell- and peptide-specific manipulations to address the individual significance of the co-localised neuronal messenger. This can for instance be achieved by cell- or gene-specific RNAi or knock-outs. Gal4-driven flp-out or CRISPR/Cas9 genome engineering is one way how this can be achieved in *Drosophila.* Further, it is important to know which neuronal messengers are co-localised. This can be achieved by immunostainings or *in situ* hybridisation. In *Drosophila,* co-localisation is facilitated due to the recent availability of appropriate transmitter-specific intragenic T2A-Gal4 lines (Lee et al., 2018; Deng et al., 2019; Kondo et al., 2020). Other appropriate methods are single-cell RNA sequencing e.g., (Allen et al., 2020) or single cell mass-spectrometric profiling (Neupert et al., 2012; Diesner et al., 2018; Diesner and Neupert, 2018). A daunting next step then is to address the effects of cocktails of co-localised neuronal messengers.
3) Peptide injections or the genetic activation of peptide signalling in *Drosophila* has provided tremendous insight into the effects of AstA and other neuropeptides. Yet, it is less clear whether these effects mimic a “natural” situation as peptide levels may or may not correspond to those in unmanipulated animals. It will therefore be important to quantitatively assess peptide release and receptor expression in unmanipulated animals, and to identify which neuronal subsets are active in a given physiological context or internal state. Methods to demonstrate the hormonal release of peptides exist, including immunoassays (RIA; ELISA, with the caveat of cross-reactions between sequence-related peptides especially in the complex haemolymph), mass-spectrometric approaches e.g., (Fastner et al., 2007), or receptor-based assays (Kim et al., 2004). These assays come with high sensitivity and specificity yet are intricate to perform, especially in small insects like fruit flies. Therefore, more efforts are needed to develop and improve new genetic tools in *Drosophila*, for instance based on visualisation of receptor activation e.g., (Inagaki et al., 2012).
4) For a deep understanding of the evolution of peptide signalling, phylogenetic sequence analysis for peptides and receptors across taxa, though important, represents only a first step. The future challenge will be to characterise the predicted ligand-receptor relationships and especially the physiological function in a representative diversity of model species. In case of AstA, for instance, it will be exciting to test whether modulation of sleep and activity is a deep function of AstA signalling in Ecdysozoa.
