# Supplementary material for: Allatostatin A Signalling: Progress and New Challenges From a Paradigmatic Pleiotropic Invertebrate Neuropeptide Family
Source: Front Physiol. 2022 Jun 24;13:920529. doi: 10.3389/fphys.2022.920529 (PMC9263205; doi:10.3389/fphys.2022.920529)
Supplement: Supplementary file 1 [file DataSheet3.docx]

**Supplementary Material 3.** AstA-induced release of digestive enzymes.

| **Species** | **amylase** | **invertase** | **protease** | **references** |
| --- | --- | --- | --- | --- |
| cockroaches | + | + | + | (Fusé et al., 1999; Aguilar et al., 2003; Sakai et al., 2006; Matsui et al., 2013) |
| crickets | + |  | + | (Woodring et al., 2009) |
| moths | +/- |  | +/- | (Lwalaba et al., 2010; Nakhaie Bahrami et al., 2018) |

Aguilar, R., Maestro, J. L., Vilaplana, L., Pascual, N., Piulachs, M.-D., and Bellés, X. (2003). Allatostatin gene expression in brain and midgut, and activity of synthetic allatostatins on feeding-related processes in the cockroach Blattella germanica. *Regul. Pept.* 115, 171–177.

Fusé, M., Zhang, J. R., Partridge, E., Nachman, R. J., Orchard, I., Bendena, W. G., et al. (1999). Effects of an allatostatin and a myosuppressin on midgut carbohydrate enzyme activity in the cockroach Diploptera punctata. *Peptides* 20, 1285–1293.

Lwalaba, D., Hoffmann, K. H., and Woodring, J. (2010). Control of the release of digestive enzymes in the larvae of the fall armyworm, Spodoptera frugiperda. *Arch. Insect Biochem. Physiol* 73, 14–29. doi: 10.1002/arch.20332.

Matsui, T., Sakai, T., Satake, H., and Takeda, M. (2013). The pars intercerebralis affects digestive activities of the American cockroach, Periplaneta Americana, via crustacean cardioactive peptide and allatostatin-A. *Journal of Insect Physiology* 59, 33–37. doi: 10.1016/j.jinsphys.2012.06.010.

Nakhaie Bahrami, M., Mikani, A., and Moharramipour, S. (2018). Effect of caffeic acid on feeding, α-amylase and protease activities and allatostatin-A content of Egyptian cotton leafworm, Spodoptera littoralis (Lepidoptera: Noctuidae). *J Pestic Sci* 43, 73–78. doi: 10.1584/jpestics.D17-086.

Sakai, T., Satake, H., and Takeda, M. (2006). Nutrient-induced alpha-amylase and protease activity is regulated by crustacean cardioactive peptide (CCAP) in the cockroach midgut. *Peptides* 27, 2157–2164. doi: 10.1016/j.peptides.2006.04.009.

Woodring, J., Diersch, S., Lwalaba, D., Hoffmann, K., and Meyering-Vos, M. (2009). Control of the release of digestive enzymes in the caeca of the cricket Gryllus bimaculatus. *Physiological Entomology* 34, 144–151. doi: 10.1111/j.1365-3032.2008.00665.x.
