# Supplementary material for: Allatostatin A Signalling: Progress and New Challenges From a Paradigmatic Pleiotropic Invertebrate Neuropeptide Family
Source: Front Physiol. 2022 Jun 24;13:920529. doi: 10.3389/fphys.2022.920529 (PMC9263205; doi:10.3389/fphys.2022.920529)
Supplement: Supplementary file 2 [file DataSheet2.docx]

**Supplementary Material 2.** Taxonomic overview of AstA-positive innervation and AstA expression in the digestive system of insects and other Ecdysozoa

| **taxon** | **foregut**  **innervation** | **midgut**  **innervation** | **hindgut**  **innervation** | **expression in EECs** | **inhibition of gut motility** | **references** |
| --- | --- | --- | --- | --- | --- | --- |
| Nematoda |  |  |  | + |  | (Nathoo et al., 2001) |
| Decapoda |  |  |  | + |  | (Yin et al., 2006) |
| Chelicerata |  |  | + |  | -(hindgut) | (Šimo and Park, 2014) |
| Dermaptera |  | + | + |  |  | (Rankin et al., 1998) |
| Blattodea |  | + | + | + | +  (fore/  hindgut) | (Lange et al., 1993; Reichwald et al., 1994; Duve et al., 1995; Lange, 1995; Veelaert et al., 1995; Yu et al., 1995; Weiß, 1997; Maestro et al., 1998; Fusé et al., 1999; Aguilar et al., 2003) |
| Orthoptera | + | + | + | + |  | (Vanden Broeck et al., 1996; Robertson and Lange, 2010; Robertson et al., 2012) |
| Heteroptera | + | + | + | + | + | (Sarkar et al., 2003; Zandawala et al., 2012; Zandawala and Orchard, 2013) |
| Hymenoptera | n.e. | n.e. | n.e. | + |  | (Christie, 2020) |
| Diptera | - | + | + | + | + (midgut) | (Duve et al., 1993; Duve H, 1994; Veenstra et al., 1995, 2008; Yoon and Stay, 1995; Hernandez-Martinez et al., 2005; Veenstra, 2009; Predel et al., 2010; Reiher et al., 2011; Zoephel et al., 2012; Robertson et al., 2014; Vanderveken and O’Donnell, 2014; Chen et al., 2016) |
| Lepidoptera | + | + | *+* | *+* | + (foregut) | (Duve et al., 1997, 1999, 2000; Abdel-latief et al., 2004; Abdel-Latief et al., 2004; Davey et al., 2005; Matthews et al., 2007; Lwalaba et al., 2010) |

Abdel-latief, M., Meyering-Vos, M., and Hoffmann, K. H. (2004). Expression and localization of the Spodoptera frugiperda allatotropin (Spofr-AT) and allatostatin (Spofr-AS) genes. *Archives of Insect Biochemistry and Physiology* 55, 188–199.

Abdel-Latief, M., Meyering-Vos, M., and Hoffmann, K. H. (2004). Type-A allatostatins from the fall armyworm, Spodoptera frugiperda: molecular cloning, expression and tissue-specific localization. *Arch. Insect Biochem. Physiol* 56, 120–132. doi: 10.1002/arch.20003.

Aguilar, R., Maestro, J. L., Vilaplana, L., Pascual, N., Piulachs, M.-D., and Bellés, X. (2003). Allatostatin gene expression in brain and midgut, and activity of synthetic allatostatins on feeding-related processes in the cockroach Blattella germanica. *Regul. Pept.* 115, 171–177.

Chen, J., Reiher, W., Hermann-Luibl, C., Sellami, A., Cognigni, P., Kondo, S., et al. (2016). Allatostatin A signalling in Drosophila regulates feeding and sleep and is modulated by PDF. *PLoS Genet.* 12, e1006346. doi: 10.1371/journal.pgen.1006346.

Christie, A. E. (2020). Assessment of midgut enteroendocrine peptide complement in the honey bee, Apis mellifera. *Insect Biochem. Mol. Biol.* 116, 103257. doi: 10.1016/j.ibmb.2019.103257.

Davey, M., Duve, H., Thorpe, A., and East, P. (2005). Helicostatins: brain-gut peptides of the moth, Helicoverpa armigera (Lepidoptera: Noctuidae). *Arch. Insect Biochem. Physiol* 58, 1–16. doi: 10.1002/arch.20020.

Duve, H., Audsley, N., and Thorpe, A. (2000). Triple co-localisation of two types of allatostatin and an allatotropin in the frontal ganglion of the lepidopteran Lacanobia oleracea (Noctuidae): innvervation and action on the foregut. *Cell and Tissue Research* 300, 153–163.

Duve, H., East, P. D., and Thorpe, A. (1999). Regulation of lepidopteran foregut movement by allatostatins and allatotropin from the frontal ganglion. *J Comp Neurol* 413, 405–416.

Duve H, J. A. H. (1994). [Hyp3]Met-callatostatin. Identification and biological properties of a novel neuropeptide from the blowfly Calliphora vomitoria. *Journal of Biological Chemistry* 269, 21059–21066.

Duve, H., Johnsen, A. H., Maestro, J. L., Scott, A. G., Crook, N., Winstanley, D., et al. (1997). Identification, tissue localisation and physiological effect in vitro of a neuroendocrine peptide identical to a dipteran Leu-callatostatin in the codling moth Cydia pomonella (Tortricidae: Lepidoptera). *Cell Tissue Res.* 289, 73–83.

Duve, H., Johnsen, A. H., Scott, A. G., Yu, C. G., Yagi, K. J., Tobe, S. S., et al. (1993). Callatostatins: neuropeptides from the blowfly Calliphora vomitoria with sequence homology to cockroach allatostatins. *Proceedings of the National Academy of Sciences* 90, 2456–2460.

Duve, H., Wren, P., and Thorpe, A. (1995). Innervation of the foregut of the cockroach Leucophaea maderae and inhibition of spontaneous contractile activity by callatostatin neuropeptides. *Physiological Entomology* 20, 33–44. doi: 10.1111/j.1365-3032.1995.tb00798.x.

Fusé, M., Zhang, J. R., Partridge, E., Nachman, R. J., Orchard, I., Bendena, W. G., et al. (1999). Effects of an allatostatin and a myosuppressin on midgut carbohydrate enzyme activity in the cockroach Diploptera punctata. *Peptides* 20, 1285–1293.

Hernandez-Martinez, S., Li, Y. P., Lanz-Mendoza, H., Rodriguez, M. H., and Noriega, F. G. (2005). Immunostaining for allatotropin and allatostatin-A and -C in the mosquitoes Aedes aegypti and Anopheles albimanus. *Cell Tissue Res.* 321, 105–113. doi: 10.1007/s00441-005-1133-5.

Lange, A. B. (1995). The effect of thirteen Dip-allatostatins on myogenic and induced contractions of the cockroach (Diploptera punctata) hindgut. *Journal of Insect Physiology* 41, 581–588.

Lange, A. B., Chan, K. K., and Stay, B. (1993). Effect of allatostatin and proctolin on antennal pulsatile organ and hindgut muscle in the cockroach, Diploptera punctata. *Archives of Insect Biochemistry and Physiology* 24, 79–92. doi: https://doi.org/10.1002/arch.940240203.

Lwalaba, D., Hoffmann, K. H., and Woodring, J. (2010). Control of the release of digestive enzymes in the larvae of the fall armyworm, Spodoptera frugiperda. *Arch. Insect Biochem. Physiol* 73, 14–29. doi: 10.1002/arch.20332.

Maestro, J. L., Bellés, X., Piulachs, M.-D., Thorpe, A., and Duve, H. (1998). Localization of allatostatin-immunoreactive material in the central nervous system, stomatogastric nervous system, and gut of the cockroach Blattella germanica. *Arch. Insect Biochem. Physiol.* 37, 269–282. doi: 10.1002/(SICI)1520-6327(1998)37:4<269::AID-ARCH2>3.0.CO;2-M.

Matthews, H. J., Audsley, N., and Weaver, R. J. (2007). Interactions between allatostatins and allatotropin on spontaneous contractions of the foregut of larval Lacanobia oleracea. *J Insect Physiol* 53, 75–83. doi: 10.1016/j.jinsphys.2006.10.007.

Nathoo, A. N., Moeller, R. A., Westlund, B. A., and Hart, A. C. (2001). Identification of neuropeptide-like protein gene families in Caenorhabditiselegans and other species. *Proc Natl Acad Sci U S A* 98, 14000–14005. doi: 10.1073/pnas.241231298.

Predel, R., Neupert, S., Garczynski, S. F., Crim, J. W., Brown, M. R., Russell, W. K., et al. (2010). Neuropeptidomics of the mosquito Aedes aegypti. *Journal of Proteome Research* 9, 2006–2015. doi: 10.1021/pr901187p.

Rankin, S. M., Stay, B., Chan, K., and Jackson, E. S. (1998). Cockroach allatostatin-immunoreactive neurons and effects of cockroach allatostatin in earwigs. *Archives of Insect Biochemistry and Physiology* 38, 155–165. doi: https://doi.org/10.1002/(SICI)1520-6327(1998)38:4<155::AID-ARCH1>3.0.CO;2-Q.

Reichwald, K., Unnithan, G. C., Davis, N. T., Agricola, H., and Feyereisen, R. (1994). Expression of the allatostatin gene in endocrine cells of the cockroach midgut. *Proc. Natl. Acad. Sci. U.S.A.* 91, 11894–11898.

Reiher, W., Shirras, C., Kahnt, J., Baumeister, S., Isaac, R. E., and Wegener, C. (2011). Peptidomics and peptide hormone processing in the Drosophila midgut. *Journal of Proteome Research* 10, 1881–1892. doi: 10.1021/pr101116g.

Robertson, L., Chasiotis, H., Galperin, V., and Donini, A. (2014). Allatostatin A-like immunoreactivity in the nervous system and gut of the larval midge Chironomus riparius: modulation of hindgut motility, rectal K+ transport and implications for exposure to salinity. *J. Exp. Biol.* 217, 3815–3822. doi: 10.1242/jeb.108985.

Robertson, L., and Lange, A. B. (2010). Neural substrate and allatostatin-like innervation of the gut of Locusta migratoria. *J. Insect Physiol.* 56, 893–901. doi: 10.1016/j.jinsphys.2010.05.003.

Robertson, L., Rodriguez, E. P., and Lange, A. B. (2012). The neural and peptidergic control of gut contraction in Locusta migratoria: the effect of an FGLa/AST. *J Exp Biol* 215, 3394–3402. doi: 10.1242/jeb.073189.

Sarkar, N. R. S., Tobe, S. S., and Orchard, I. (2003). The distribution and effects of Dippu-allatostatin-like peptides in the blood-feeding bug, Rhodnius prolixus. *Peptides* 24, 1553–1562.

Šimo, L., and Park, Y. (2014). Neuropeptidergic control of the hindgut in the black-legged tick Ixodes scapularis. *Int J Parasitol* 44, 819–826. doi: 10.1016/j.ijpara.2014.06.007.

Vanden Broeck, J., Veelaert, D., Bendena, W. G., Tobe, S. S., and De Loof, A. (1996). Molecular cloning of the precursor cDNA for schistostatins, locust allatostatin-like peptides with myoinhibiting properties. *Mol. Cell. Endocrinol.* 122, 191–198.

Vanderveken, M., and O’Donnell, M. J. (2014). Effects of diuretic hormone 31, drosokinin, and allatostatin A on transepithelial K^+^ transport and contraction frequency in the midgut and hindgut of larval Drosophila melanogaster. *Arch. Insect Biochem. Physiol.* 85, 76–93. doi: 10.1002/arch.21144.

Veelaert, D., Schoofs, L., Tobe, S. S., Yu, C. G., Vullings, H. G., Couillaud, F., et al. (1995). Immunological evidence for an allatostatin-like neuropeptide in the central nervous system of Schistocerca gregaria, Locusta migratoria and Neobellieria bullata. *Cell Tissue Res* 279, 601–611.

Veenstra, J. A. (2009). Peptidergic paracrine and endocrine cells in the midgut of the fruit fly maggot. *Cell Tissue Res* 336, 309–323. doi: 10.1007/s00441-009-0769-y.

Veenstra, J. A., Agricola, H.-J., and Sellami, A. (2008). Regulatory peptides in fruit fly midgut. *Cell Tissue Res* 334, 499–516. doi: 10.1007/s00441-008-0708-3.

Veenstra, J. A., Lau, G. W., Agricola, H.-J., and Petzel, D. H. (1995). Immunohistological localization of regulatory peptides in the midgut of the female mosquitoAedes aegypti. *Histochem Cell Biol* 104, 337–347. doi: 10.1007/BF01458127.

Weiß, T. (1997). *Untersuchungen zur Lokalisation und Funktion von Neuropeptiden im Darmsystem der Schabe Periplaneta americana (L.).* Dissertation Friedrich-Schiller-Universität, Jena.

Yin, G.-L., Yang, J.-S., Cao, J.-X., and Yang, W.-J. (2006). Molecular cloning and characterization of FGLamide allatostatin gene from the prawn, Macrobrachium rosenbergii. *Peptides* 27, 1241–1250. doi: 10.1016/j.peptides.2005.11.015.

Yoon, J. G., and Stay, B. (1995). Immunocytochemical localization of Diploptera punctata allatostatin-like peptide in Drosophila melanogaster. *Journal of Comparative Neurology* 363, 475–488.

Yu, C., Stay, B., Ding, Q., Bendena, W., and Tobe, S. (1995). Immunochemical identification and expression of allatostatins in the gut. *J. Insect Physiol.* 41, 1035–1043. doi: 10.1016/0022-1910(95)00075-6.

Zandawala, M., Lytvyn, Y., Taiakina, D., and Orchard, I. (2012). Cloning of the cDNA, localization, and physiological effects of FGLamide-related allatostatins in the blood-gorging bug, Rhodnius prolixus. *Insect Biochemistry and Molecular Biology* 42, 10–21. doi: 10.1016/j.ibmb.2011.10.002.

Zandawala, M., and Orchard, I. (2013). Post-feeding physiology in Rhodnius prolixus: The possible role of FGLamide-related allatostatins. *General and Comparative Endocrinology* 194, 311–317. doi: 10.1016/j.ygcen.2013.10.005.

Zoephel, J., Reiher, W., Rexer, K.-H., Kahnt, J., and Wegener, C. (2012). Peptidomics of the agriculturally damaging larval stage of the cabbage root fly Delia radicum (Diptera: Anthomyiidae). *PLoS ONE* 7, e41543. doi: 10.1371/journal.pone.0041543.
