# Supplementary material for: Allatostatin A Signalling: Progress and New Challenges From a Paradigmatic Pleiotropic Invertebrate Neuropeptide Family
Source: Front Physiol. 2022 Jun 24;13:920529. doi: 10.3389/fphys.2022.920529 (PMC9263205; doi:10.3389/fphys.2022.920529)
Supplement: Supplementary file 3 [file DataSheet1.docx]

Supplementary material 1

AstA and AstA-like peptide sequences used to produce the consensus sequences in Fig. 1a)in FASTA format.

>Platynereis_dumerilii_AstA1_1

KMIDYGLKFHGNG

>Platynereis_dumerilii_AstA1_2

QTDNTLKFYGPG

>Platynereis_dumerilii_AstA1_3

TDRARSGIKMRLSG

>Platynereis_dumerilii_AstA1_4

VDDISKFSGLG

>Platynereis_dumerilii_AstA1_5

DDNIFRFSELG

>Platynereis_dumerilii_AstA1_6

VNNALKFSGLG

>Platynereis_dumerilii_AstA1_7

TNDALEFSGLG

>Platynereis_dumerilii_AstA1_8

GNDALQFSGLG

>Platynereis_dumerilii_AstA2_1

ANNALKGSGLG

>Platynereis_dumerilii_AstA2_2

NDALKFSGLG

>Platynereis_dumerilii_AstA2_3

ANDALKFSGLG

>Platynereis_dumerilii_AstA2_4

EIDTLKFSELG

>Platynereis_dumerilii_AstA2_5

NDALKFSGLG

>Platynereis_dumerilii_AstA2_6

TEVGLRFSGLG

>Platynereis_dumerilii_AstA2_7

NDDIMNFSGLG

>Platynereis_dumerilii_AstA2_8

ANDVMTFSGLG

>Platynereis_dumerilii_AstA2_9

MDETLKFSGVG

>Platynereis_dumerilii_AstA2_10

SDNGLKFSGLG

>Platynereis_dumerilii_AstA2_11

ANEIFRLSGLG

>Platynereis_dumerilii_AstA2_12

VNSMHKSGPG

>Platynereis_dumerilii_AstA2_13

LSVDPGISLIGLG

>Lottia_gigantea_Buccalin1

GMDKFGFAGGVG

>Lottia_gigantea_Buccalin2

GLDKFGFTGQLG

>Lottia_gigantea_Buccalin3

DMDSFGFAGQLG

>Lottia_gigantea_Buccalin4

GLDQYGFTGQLG

>Lottia_gigantea_Buccalin5

GLDQYGFAGQLG

>Lottia_gigantea_Buccalin6

GLDHYGFAGQLG

>Lottia_gigantea_Buccalin7

GFDQFGFAGQLG

>Lottia_gigantea_Buccalin8

GLDQLGFTGQLG

>Lottia_gigantea_Buccalin9

QMDIFGYRGQLG

>Lottia_gigantea_Buccalin10

QSIDKYSFLGAGIG

>Mytilus_galloprovincialis_Buccalin1a

KMDMYRFHGSLG

>Mytilus_galloprovincialis_Buccalin1b

KIDYARFLGSLG

>Mytilus_galloprovincialis_Buccalin1c

SKYDRYMFAPSLG

>Mytilus_galloprovincialis_Buccalin1d

RLDRFSYFGNLG

>Mytilus_galloprovincialis_Buccalin1e

GMDKLSYFGSLG

>Mytilus_galloprovincialis_Buccalin1f

GLDRLSFFGGLG

>Mytilus_galloprovincialis_Buccalin1g

RMDRLSYFGGLG

>Mytilus_galloprovincialis_Buccalin1h

GIDKYSLFGSLG

>Mytilus_galloprovincialis_Buccalin1i

SIDQDRLQEILG

>Mytilus_galloprovincialis_Buccalin2a

LFERLASGLVG

>Mytilus_galloprovincialis_Buccalin2b

PFDRLASGLVG

>Mytilus_galloprovincialis_Buccalin2c

EFDRLASGLVG

>Mytilus_galloprovincialis_Buccalin2d

MDPLASSLVG

>Caenorhabditis_elegans_AstA1

MAAPKQMVFGFG

>Caenorhabditis_elegans_AstA2

YKPRSFAMGFG

>Caenorhabditis_elegans_AstA3

AAMRSFNMGFG

>Caenorhabditis_elegans_nlp6_b

LIMGLG

>Caenorhabditis_elegans_nlp5_1

SVSQLNQYAGFDTLGGMGLG

>Caenorhabditis_elegans_nlp5_2

ALSTFDSLGGMGLG

>Caenorhabditis_elegans_nlp5_3

ALQHFSSLDFTLGGMGFG

>Pristionchus_pacificus_AstA1

SDPRMFSAAFG

>Pristionchus_pacificus_AstA2

GDPRMFSSAFG

>Ixodes_scapularis_AstA1

RPPAAMYGFGLG

>Ixodes_scapularis_AstA2

GERPQHPLRYGFGLG

>Ixodes_scapularis_AstA3

ERHRFGFGLG

>Ixodes_scapularis_AstA4

RYNFGLG

>Stegodyphus_mimosarum_AstA_1

VPQPYGFGLG

>Stegodyphus_mimosarum_AstA_2

DGNFERFGFGLG

>Stegodyphus_mimosarum_AstA_3

AEPSRFTFGLG

>Stegodyphus_mimosarum_AstA_4

GEHNRFQFGLG

>Stegodyphus_mimosarum_AstA_5

EDDHSRFAFGLG

>Stegodyphus_mimosarum_AstA_6

GPEDNRFAFGLG

>Stegodyphus_mimosarum_AstA_7

GPEDQRFAFGLG

>Stegodyphus_mimosarum_AstA_8

AAEDNRFAFGLG

>Stegodyphus_mimosarum_AstA_9

ASEENRFAFGLG

>Stegodyphus_mimosarum_AstA_10

GPEESRFAFGLG

>Stegodyphus_mimosarum_AstA_11

GPEENRFAFGLG

>Stegodyphus_mimosarum_AstA_12

DDHNRFAFGLG

>Stegodyphus_mimosarum_AstA_13

DDDRSRFAFGLG

>Stegodyphus_mimosarum_AstA_14

SDEHNRFAFGLG

>Stegodyphus_mimosarum_AstA_15

FSFGLG

>Acanthoscurria_geniculata_AstA_1

SPQLYSFGLG

>Acanthoscurria_geniculata_AstA_2

DGNFERYSFGLG

>Acanthoscurria_geniculata_AstA_3

AVEPSRFAFGLG

>Acanthoscurria_geniculata_AstA_4

GDSPSSRFSFGLG

>Acanthoscurria_geniculata_AstA_5

MEQRYAFGLG

>Acanthoscurria_geniculata_AstA_6

EPSRYAFGLG

>Acanthoscurria_geniculata_AstA_7

FSFGLG

>Acanthoscurria_geniculata_AstA_8

AVDQRFAFGLG

>Daphnia_pulex_AstA_1

SFGGNPTGDPNLNIYSFGLG

>Daphnia_pulex_AstA_2

TSRSYSINPYSFGLG

>Daphnia_pulex_AstA_3

GGNAKSYPQQIPYSFGLG

>Daphnia_pulex_AstA_4

NPTKYNFGLG

>Daphnia_pulex_AstA_5

PDRFGFGLG

>Daphnia_pulex_AstA_6

LPVYNFGLG

>Homarus_americanus_AstA_1

EPYAFGLG

>Homarus_americanus_AstA_2

SPYAFGLG

>Homarus_americanus_AstA_3

SGPYAFGLG

>Homarus_americanus_AstA_4

SGPYSFGLG

>Homarus_americanus_AstA_5

ASPYAFGLG

>Homarus_americanus_AstA_6

AGPYAFGLG

>Homarus_americanus_AstA_7

VGPYAFGLG

>Homarus_americanus_AstA_8

TPSYAFGLG

>Homarus_americanus_AstA_9

SQYTFGLG

>Homarus_americanus_AstA_10

AGGAYSFGLG

>Periplaneta_americana_AstA1

LYDFGLG

>Periplaneta_americana_AstA2

LPVYNFGLG

>Periplaneta_americana_AstA3

SKMYGFGLG

>Periplaneta_americana_AstA4

SGNDGRLYSFGLG

>Periplaneta_americana_AstA5

DRMYSFGLG

>Periplaneta_americana_AstA6

ARPYSFGLG

>Periplaneta_americana_AstA7

SPSGMQRLYGFGLG

>Periplaneta_americana_AstA8

GGSMYSFGLG

>Periplaneta_americana_AstA9

ADGRLYAFGLG

>Periplaneta_americana_AstA10

PVSSARQTGSRFNFGLG

>Periplaneta_americana_AstA11

SPQGHRFSFGLG

>Carausius_morosus_AstA1

LYDFGLG

>Carausius_morosus_AstA2

LPVYNFGLG

>Carausius_morosus_AstA3

SDSRQYSFGLG

>Carausius_morosus_AstA4

GRQYSFGLG

>Carausius_morosus_AstA5

TKPYSFGLG

>Carausius_morosus_AstA6

TSSLYSFGLG

>Carausius_morosus_AstA7

AEKPHSLYSFGLG

>Carausius_morosus_AstA8

ADGRMYAFGLG

>Carausius_morosus_AstA9

PADETSRHSGHRFGFGLG

>Carausius_morosus_AstA10

SQHRFSFGLG

>Carausius_morosus_AstA11

SLQYPFAIG

>Carausius_morosus_AstA12

RPYNFGLG

>Carausius_morosus_AstA13

IPMYDFGLG

>Carausius_morosus_AstA14

TSSGQRSLYSFGLG

>Carausius_morosus_AstA15

AERPHSMYSFGLG

>Acyrthosiphon_pisum_AstA1

AHKQYGFGLG

>Acyrthosiphon_pisum_AstA2

LYRQYEFGLG

>Acyrthosiphon_pisum_AstA3

SASKQYGFGLG

>Acyrthosiphon_pisum_AstA4

AALKQYEFGLG

>Acyrthosiphon_pisum_AstA5

ASPTFYSFGLG

>Acyrthosiphon_pisum_AstA6

ASPQYSFGLG

>Acyrthosiphon_pisum_AstA7

TADDMGHGQRFAFGLG

>Acyrthosiphon_pisum_AstA8

ARLQYGFGLG

>Cimex_lectularius_AstA1

LYDFGLG

>Cimex_lectularius_AstA2

LPVYNFGLG

>Cimex_lectularius_AstA3

AAGEKTYSFGLG

>Cimex_lectularius_AstA4

GRQYAFGLG

>Cimex_lectularius_AstA5

LPKQYSFGLG

>Cimex_lectularius_AstA6

PEGKMYSFGLG

>Cimex_lectularius_AstA7

TKSGKDLRYMFGIG

>Cimex_lectularius_AstA8

DVDEDERARQERSMHYNFGLG

>Camponotus_floridanus_AstA1

LPLYNFGIG

>Camponotus_floridanus_AstA2

TRPFSFGIG

>Camponotus_floridanus_AstA3

LRDYRFGIG

>Camponotus_floridanus_AstA4

GGQPFSFGIG

>Apis_mellifera_AstA1

LPVYNFGIG

>Apis_mellifera_AstA2

GRDYSFGLG

>Apis_mellifera_AstA3

GRQPYSFGLG

>Apis_mellifera_AstA4

PNDMLSQRYHFGLG

>Agrotis_ipsilon_AstA1

SPHYDFGLG

>Agrotis_ipsilon_AstA2

LPVYNFGLG

>Agrotis_ipsilon_AstA3

SRPYSFGLG

>Agrotis_ipsilon_AstA4

ARPYSFGLG

>Agrotis_ipsilon_AstA5

ARAYDFGLG

>Agrotis_ipsilon_AstA6

LPLYNFGLG

>Agrotis_ipsilon_AstA7

ARSYNFGLG

>Agrotis_ipsilon_AstA8

LASKFNFGLG

>Agrotis_ipsilon_AstA9

ERDMHRFSFGLG

>Aedes_aegypti_AstA1

SPKYNFGLG

>Aedes_aegypti_AstA2

LPHYNFGLG

>Aedes_aegypti_AstA3

ASAYRYHFGLG

>Aedes_aegypti_AstA4

RVYDFGLG

>Aedes_aegypti_AstA5

LPNRYNFGLG

>Drosophila_melanogaster_AstA1

VERYAFGLG

>Drosophila_melanogaster_AstA2

LPVYNFGLG

>Drosophila_melanogaster_AstA3

SRPYSFGLG

>Drosophila_melanogaster_AstA4

TTRPQPFNFGLG
